# Strain-level variations of *Dirofilaria immitis* microfilariae in two biochemical assays

**DOI:** 10.1371/journal.pone.0307261

**Published:** 2024-07-17

**Authors:** Naomi Hampton, Vicki Smith, Matthew T. Brewer, Jeba R. J. Jesudoss Chelladurai

**Affiliations:** 1 Department of Diagnostic Medicine/Pathobiology, Kansas State University College of Veterinary Medicine, Manhattan, KS, United States of America; 2 Department of Veterinary Pathology, Iowa State University College of Veterinary Medicine, Ames, Iowa, United States of America; University of California Riverside, UNITED STATES OF AMERICA

## Abstract

**Background:**

The increase in reports of resistance to macrocyclic lactones in the canine heartworm, *Dirofilaria immitis* is alarming. While DNA based tests have been well-validated, they can be expensive. In a previous study, we showed that two biochemical tests adapted to a 96- well plate format and read in a spectrophotometer could detect differences among lab validated *D*. *immitis* isolates. The two tests- Resazurin reduction and Hoechst 33342 efflux—detect metabolism and P-glycoprotein activity respectively in microfilariae isolated from infected dog blood.

**Methods:**

Our objective was to optimize the two assays further by testing various assay parameters in *D*. *immitis* isolates not tested previously. We tested microfilarial seeding density, incubation time and the effect of in vitro treatment with ivermectin and doxycycline in five other *D*. *immitis* isolates—JYD-34, Big Head, Berkeley, Georgia III and LOL. All assays were performed in 3 technical replicates and 2–4 biological replicates. To understand the molecular basis of the assays, we also performed qPCR for selected drug metabolism and elimination associated genes of the ABC transporter and cytochrome P450 gene families.

**Results:**

Metabolism and ABC transporter activity as detected by these assays varied between strains. Anthelmintic status (resistant or susceptible) did not correlate with metabolism or P-gp efflux. Basal transcriptional variations were found between strains in ABC transporter and cytochrome P450 genes.

**Conclusions:**

These assays provide a greater understanding of the biochemical variation among isolates of *D*. *immitis*, which can be exploited in the future to develop *in vitro* diagnostic tests capable of differentiating susceptible and resistant isolates.

## Background

*Dirofilaria immitis* is a mosquito-borne filarial nematode and the causative agent of canine and feline heartworm disease. Adult *D*. *immitis* worms reside in the pulmonary arteries of dogs and cats and can cause severe cardiopulmonary disease resulting in death unless promptly diagnosed and treated. Adult female worms produce microfilariae (a pre-larval stage), which are released into blood. Microfilariae must be ingested by female mosquitoes and undergo development to the third larval (L3) stage within the mosquito before they can be transmitted to susceptible dogs. L3 stages, after deposition by biting female mosquitoes, undergo development to the fourth larval (L4) stage in the subcutaneous tissues of dogs and cats. *Dirofilaria immitis* control relies on the regularly timed administration of one of several drugs from the macrocyclic lactone drug class, labelled as “heartworm disease preventatives”. Depending on the product, these drugs are FDA-labelled for monthly or biannual or annual use. These kill the infectious L3 and early L4 stages of the worm within the subcutaneous tissue of dogs, and can sometimes but not always be microfilaricidal at labelled doses [1].

The efficacy of the preventative drugs at registration was 100%. In the last two decades, however, there have been several instances of “loss of effectiveness (LOE)” of the macrocyclic lactone preventatives in some isolates of *D*. *immitis* in the United States [2]. LOE can be due to two separate causes, which are not easily differentiable in clinical situations: (a) the result of improper owner compliance in the administration of macrocyclic lactone drugs, or (b) true resistance in the L3 and/or L4 stages of *D*. *immitis* [3]. In the latter case, infectious L3s survive the effect of macrocyclic lactone drugs and develop to adult worms. Clinically, a presumptive diagnosis of drug resistance can be made using the 7-day microfilarial suppression test (MFST) [4,5]. The current gold standard to obtain a confirmatory diagnosis is experimentally infecting and treating dogs [6,7].

Several named “strains” have been partially or fully characterized for macrocyclic lactone susceptibility—JYD-34 [8], ZoeMO [9] etc. and have also been used in FDA-drug approval studies. While “ML resistant” refers to the inability of the drug to kill the L3 stage within the host, infectious L3s (iL3s) which develop within mosquitoes are hard to isolate in large quantities for research and screening assays. Typically, microfilariae isolated from the blood of experimentally infected dogs are used in *in vitro* investigations. The major goal of some of these investigations have been to differentiate between resistant and susceptible isolates. These assays have included SNP assessments [10–12], microsatellite analyses [13], whole genome analyses [14], microfilarial motility tests [15], larval migration inhibition assays [16], leukocyte binding assays [17], biochemical assays [18] and enzyme assays such as for triosephosphate isomerase (TPI) [19]. These tests were not completely discriminatory between resistant and susceptible isolates in all cases, with SNP testing showing the most promise.

In a previous study, we evaluated seven biochemical assays with four laboratory validated strains of *D*. *immitis* microfilariae (Missouri, JYD-27, Metairie and Yazoo) exposed to macrocyclic lactones *in vitro* [18]. These tests were based on altered membrane permeability [20], metabolic activity [21], or P-glycoprotein-related efflux activity [22] as surrogate measures of macrocyclic lactone susceptibility. Interestingly, the resazurin reduction assay (based on metabolic activity) showed significant constitutive differences between strains even in the absence of macrocyclic lactones suggesting constitutive differences in the redox potential of the strains. Additionally, the Hoechst 33342 efflux assay (based on P-glycoprotein activity)revealed constitutive differences between strains. However, only a single concentration of microfilariae (300 microfilariae/well) and 4 strains had been tested in the previous study. In this study, we sought to optimize these assays by testing the effects of microfilarial seeding density, incubation time and drugs on five *D*. *immitis* strains not previously tested (Berkeley, Big Head, Georgia III, JYD-34 and LOL) and to assess repeatability among different blood collections. We also aimed to assess constitutive gene expression of candidate ABC transporters and cytochrome P450s that could explain the phenotypic results observed in the two biochemical assays.

## Methods

### Ethics

All experiments performed in this study were approved by the Institutional Biosafety Committee at Kansas State University under protocol 1556-VCS.

### Parasites

*D*. *immitis* microfilariae in canine blood collected in EDTA tubes were purchased from TRS Labs (USA) and shipped overnight to Kansas State University. All studies described were carried out between May and August 2021.

Nomenclature rules of strains have been described and includes name of the isolate, passage number and treatments [13]. However, except in controlled experimental infections, the exact passage number of the strain is not known/easily available. In this paper, we use the old strain nomenclature without passage names. Five *D*. *immitis* strains were used in this study:

1. JYD-34 (ML-resistant): The JYD-34 strain was originally isolated from a dog in Illinois in 2010 and has been maintained at TRS Labs. Ivermectin, milbemycin oxime and selamectin had <100% efficacy against this strain [8]. JYD-27 and JYD-34 are different isolates from the same infected dog [17].
2. Berkeley (ML-susceptible): The Berkeley strain was originally isolated from South Carolina in 2014 and has been maintained at TRS Labs [23].
3. Big Head (ML-susceptible): The Big Head strain was originally isolated from a Labrador Retriever dog in Louisiana in 2015 [24].
4. Georgia III (ML-susceptible): The Georgia III strain was originally isolated from Oconee County, GA in 2017 [19].
5. LOL (ML-status unknown): The LOL strain was originally isolated from an unknown source and is maintained at TRS Labs.

### Parasite isolation

Microfilariae in blood were isolated using the protocol described previously [18]. Briefly, blood tubes were gently mixed and 1 mL of blood was transferred to a sterile tube with 10 mL of 0.2% saponin solution. The tubes were mixed by inversion, incubated at 37°C for 30 minutes and centrifuged at 500 xG for 10 minutes. The supernatant was discarded, the pellet was washed twice with 1x Dulbecco’s PBS and resuspended in 2 mL of prewarmed sterile RPMI-1640. Microfilariae present in three 10 μL aliquots of the suspension were enumerated, ensuring that only motile microfilariae were counted, and averaged to obtain the average microfilarial count in the suspension. Microfilariae were freshly isolated for each day of the study. All assays were carried out within 3 days of receiving blood samples. Blood was stored at 4°C after arrival and in between testing.

### Assays—resazurin assay and Hoechst 33342 assay

Dilutions of microfilariae were made in RPMI-1640 from enumerated “stock” suspensions. Microfilariae were plated in technical triplicates in 96-well flat-bottomed plates at different concentrations to obtain 50, 100, 300 or 500 microfilariae/well in a final volume of 100 μL. No microfilariae (dye only) controls in triplicate were used as control in every 96-well plate. Each assay was repeated a minimum of 2 times (average of 3 times; maximum 4 times) with each strain to represent biological replicates (biological replicates because microfilariae are a genetic admixture of organisms derived from polyandrous parents). At least two strains were tested on each plate simultaneously.

For the resazurin reduction assay, microfilariae in wells were incubated with 20 μL of resazurin dye (Promega) at 37°C in darkness. Resazurin has low intrinsic fluorescence until it is reduced to resorufin, a highly fluorescent compound. For the Hoechst 33342 assay, microfilariae in wells were incubated with 20 μL of 20 μM concentration of Hoechst 33342 dye (Thermo Scientific) at 37°C in darkness. Incubation period is described below.

Plate effects were carefully controlled for including (a) edge effects, by avoiding edge wells when possible, (b) positional effects in the reader, by inverting the plate and obtaining a second read and (c) potential volumetric errors, by using the “path check” feature of the spectrophotometer to normalize for minor variations in well volumes.

### Parameters tested

#### Incubation times

To assess optimal timing, two strains–*D*. *immitis* Big Head and JYD-34 were tested across multiple incubation times. Dyes (resazurin and Hoechst 33342) were incubated with the 50 to 500 microfilariae of Big Head or JYD-34 for 10 mins, 1 hour, 2 hours or 24 hours prior to spectrophotometric reads. These readings were performed on the same wells/plates. Plates were held at 37°C in darkness in between reads.

#### Mode of detection

For the resazurin assay, fluorescence was measured in a BioTek Synergy H1 Hybrid spectrophotometer at an Ex./Em. of 579nm/584nm. Absorbance was measured in a BioTek Epoch spectrophotometer at 650nm (resazurin) and 573nm (resorufin). For Hoechst 33342 assay, fluorescence was measured in a BioTek Synergy H1 Hybrid spectrophotometer at an Ex./Em. of 361nm/486nm. All wavelengths were in accordance with the dye manufacturer’s recommendations.

#### Microfilarial concentrations

After optimization of incubation time and mode of detection, an optimized incubation time of 1 hour was used and 50 to 500 microfilariae from all strains were tested for resazurin reduction and Hoechst 33342 efflux (Table 1).

**Table 1:** Assay optimization parameters.

Parameter	Conditions tested	Strain tested	Optimized condition
**Mode of detection—Resazurin**	Fluorescence Absorbance	Big Head, JYD-34	Fluorescence (Ex-361nm/Em-486nm)
**Incubation time**	10 mins, 1 hour, 2 hours or 24 hours	Big Head, JYD-34	1 hour
**Microfilarial concentrations**	50 to 500 microfilariae/well	Big Head, JYD-34, Berkeley, LOL, Georgia III	≥ 300 microfilariae per well
**Drugs**	Ivermectin ± Doxycycline	Big Head, JYD-34, Berkeley, LOL, Georgia III	-

#### Drugs

Three hundred microfilariae (optimized concentration/well) from each of the 5 strains were incubated for 1 hour with ivermectin (final concentration of 3.54 nM), doxycycline HCl (final concentration of 56.5 μM) or a combination of ivermectin and doxycycline following [25] in a final concentration of 0.06% DMSO. Dye only (no microfilariae) and 0.06% DMSO were used as negative controls. Each drug was tested with each strain in technical triplicates in a minimum of 3 replicates.

#### RNA extraction and qPCR

Microfilariae isolated using the saponin method and not used in the biochemical assays were centrifuged at 500xG for 10 minutes, suspension media was removed and stored as pellets without any additional preservatives at -80°C until RNA extraction. RNA was extracted using the Trizol method according to the manufacturer’s recommendation and quantified using Nanodrop spectrophotometer. Complementary DNA was synthesized from RNA using the iScript cDNA kit (BioRad). qPCR was performed with PowerTrack SYBR Green Master Mix (Thermo) using primers described by Lucchetti et al. [25] and custom designed primers (Table 2). *Dirofilaria immitis* 18S was used as the housekeeping gene. Relative expression was determined using the 2^-ΔΔCt^ method.

**Table 2:** Primers used in this study.

Gene	Primer name	Primer sequence	GenBank Accession	Reference
***18S* rDNA**	18SQ-F	GGGACAAGCGGTGTTTAGC		[25]
	18SQ-R	GCACGCTGATTCCTCCAGT		
**Dim-Pgp-3**	DimPgp3_F1	TGCGAGCATACGATGAAGCA	KP296249.1	Custom designed
	DimPgp3_R1	AGCAACTTCGGCATACTGTG		
**Dim*-*Pgp-10**	DimmScaf48-cDNA-F8	GCCATCGTAGGTCCATCAGGTTCTGGT		[25]
	DimmScaf48-cDNA-R12	TGTTCAACTGAAACGACCACACGTC		
**Dim*-*Pgp-11**	DimmScaf04-cDNA-F6	TTAACAGTGTTGATGAAGGATCAAATCC		[25]
	DimmScaf04-cDNA-R6	ATATTTCGCTGCGGTCTTGTTGG		
**Dim-haf-1**	Dimhaf1_F1	ACTATCGGTGCTCGTGCTTT	KP296251.1	Custom designed
	Dimhaf1_R1	CCTGAGGTAACAATTGCCGC		
**Dim*-haf-*4**	DimmScaf101-cDNA-F5	GTGCAAACTCGAGGTTTTGCTGT		[25]
	DimmScaf101-cDNA-R2	TCCACCTCGAAGACCTCCAGCA		
**Dim-*haf*-5.1**	Dimhaf5var1_F1	ATGTTTGGGATCGCAGCGT	KP296257.1	Custom designed
	Dimhaf5var1_R1	TGTGCAACGCGTGCAAATAAT		
**DimCYP4V2**	DimCYP450_4v2_F1	GGACAACGATTCGCACTTATGG	JABVXT010000009	Custom designed
	DimCYP450_4v2_R1	GCGCATCTGATCTGTACGAA		
**DimCYP2B19**	DimCYP450_2b19_F1	ATGCATGCCGTTTGTGTAGG	JABVXT010000012.1	Custom designed
	DimCYP450_2b19_R1	CCGCCACGGTCCGTAATAAA		

### Statistical analyses

All datasets were compiled in GraphPad Prism (version 9.5). Resazurin and Hoechst 33342 data (Relative fluorescence units (RFU)) were baseline corrected to the no microfilariae control in each replicate plate. Baseline correction used the formula: (RFU of well- RFU of dye only value from the same plate). Scatter plots with linear regression were plotted in R version 4.2.2 for raw RFU and baseline corrected data using *ggplot2* version 3.4.1 [26] and *ggprism* version 1.0.4 [27]. Correlation coefficients with p-values were annotated with *ggpubr* version 0.6.0 [28]. R^2^ values of the linear regression were annotated with *ggpmisc* version 0.5.2 [29]. ANCOVA with Tukey’s multiple comparison was performed using *multcomp* version 1.4.23 [30]. All R analyses were performed in the RStudio IDE build 492. Graphs for drug assays and relative gene expression were created in GraphPad Prism (version 9.5).

## Results

### Incubation times—Resazurin reduction assay

**Fig 1:**
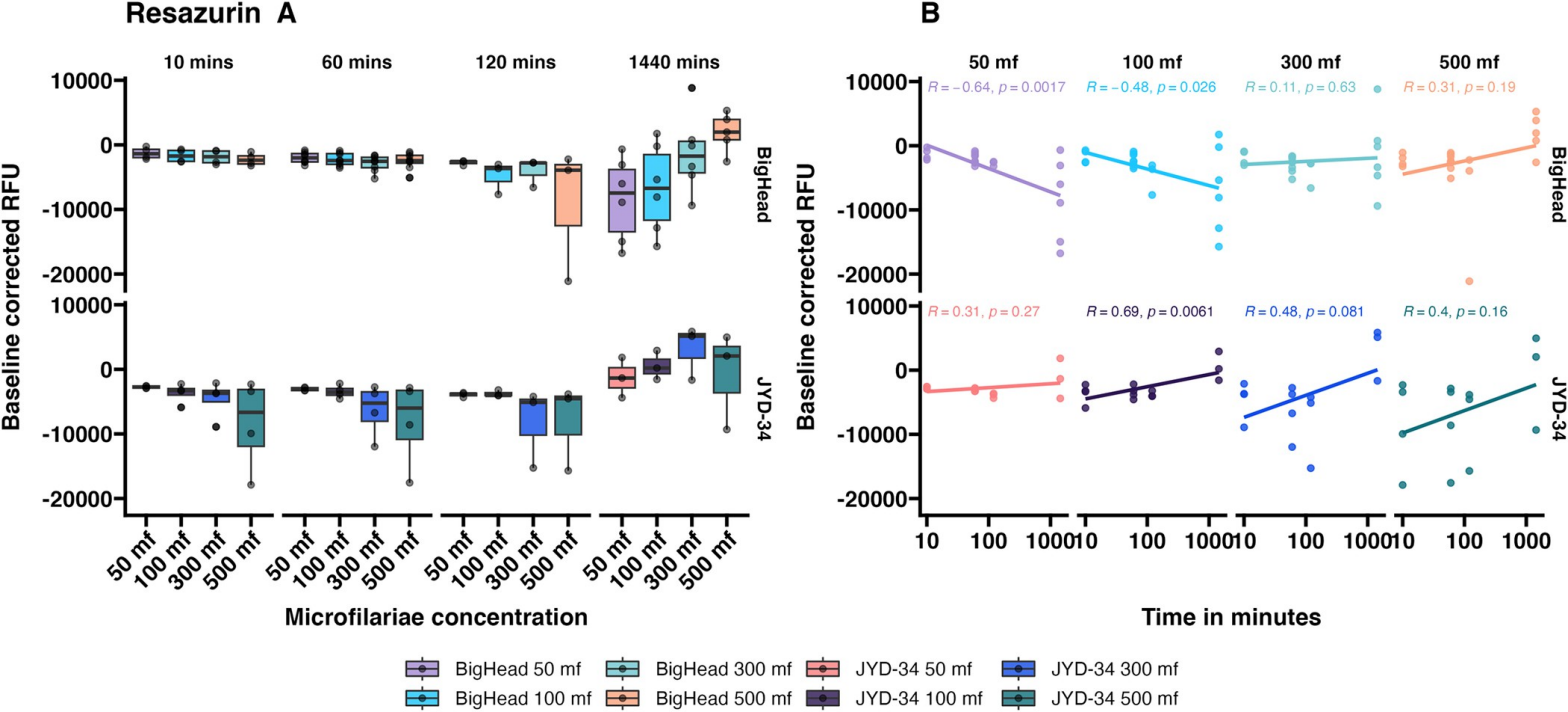
Baseline corrected fluorescence values (mean ± SE) obtained by incubating resazurin with *D*. *immitis* microfilariae of two different strains (JYD-34 and Big Head) at four different concentrations (50 to 500 microfilaria per well) for incubation periods ranging from 10 minutes to 24 hours at 37°C. Panel A: Values arranged by incubation time. Panel B. Values arranged by microfilariae concentration. Correlation values and p-values are shown in panel B.

To optimize incubation times, four concentrations of JYD-34 and Big Head strain microfilariae were incubated with resazurin (Fig 1, S1 Fig in S1 File) at four incubation times– 10 minutes, 1 hour, 2 hours or 24 hours. Non-fluorescent resazurin is reduced to fluorescent resorufin during incubation, and fluorescence of resorufin was measured at the end of incubation at Ex./Em. of 579nm/584nm. We hypothesized that resazurin metabolism to resorufin would increase with time. Raw relative fluorescence values of resorufin were measured and found to increase with incubation time in both strains at all microfilarial concentrations (S1 Fig in S1 File). However, when controlled for baseline resazurin dye fluorescence differences in resazurin reduction over time in each strain and time point were not significantly different (Fig 1A) (ANCOVA with Tukey contrasts; model: Baseline controlled value ~ Time in Minutes + Strain + microfilaria concentration; adj. p>0.05). Variability was low at the 10- and 60-minute incubation times. Interestingly, resazurin reduction increased with time at microfilarial concentrations of 300 and 500 microfilariae in both strains (as hypothesized) but decreased at 50 and 100 microfilariae in the Big Head strain (Fig 1B). Thus, a minimum of 300 microfilariae was established as the minimum necessary for the assay. Correlations between the microfilarial concentrations and incubation time was higher in the JYD-34 strain than in Big Head strain (Fig 1B). However, differences were not significant between the two strains (ANCOVA with Tukey contrasts; adj. p>0.05) or between microfilarial concentrations (ANCOVA with Tukey contrasts; adj. p>0.05).

### Incubation times—Hoechst 33342 efflux assay

**Fig 2:**
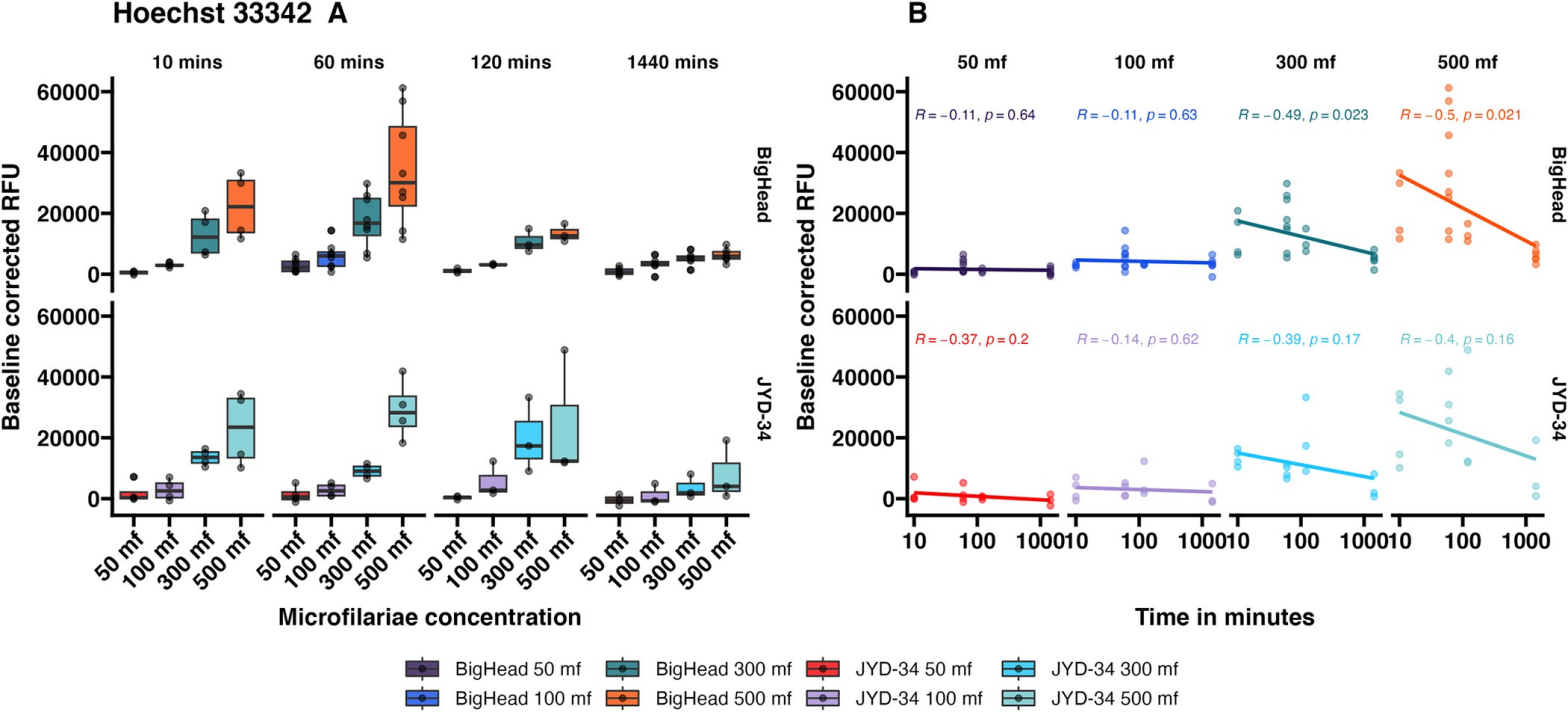
Baseline corrected fluorescence values (mean ± SE) obtained by incubating Hoechst 33342 with *D*. *immitis* microfilariae of two different strains (JYD-34 and Big Head) at four different concentrations (50 to 500 microfilaria per well) for incubation periods ranging from 10 minutes to 24 hours at 37°C. Panel A: Values arranged by incubation time. Panel B. Values arranged by microfilariae concentration. Correlation values and p-values are shown in panel B.

To optimize incubation times, four concentrations of JYD-34 and Big Head strain microfilariae were incubated with Hoechst 33342 (Fig 2, S2 Fig in S1 File) at four incubation times– 10 minutes, 1 hour, 2 hours or 24 hours. Hoechst 33342 assays are based on the principle of dye efflux and a lower fluorescence value is expected over time if the dye is effluxed. We hypothesized that Hoechst 33342 accumulation in microfilariae would decrease with time due to dye efflux by ABC transporters. The dye fluoresces only within biological membranes but not in aqueous solutions. Raw relative fluorescence values of Hoechst 33342 were measured and found to have decreased with incubation time in both strains when 300 and 500 total microfilariae/well were used (negative correlation coefficients) (S2 Fig in S1 File).

When controlled for baseline dye fluorescence, in overall comparisons, there were differences in Hoechst 33342 efflux between 10 minutes and 24 hours (ANCOVA with Tukey contrasts; model: Baseline controlled value ~ Time in Minutes + Strain + microfilaria concentration; adj. p < 0.01), 1 hour and 24 hours (ANCOVA with Tukey contrasts; adj. p < 0.001) and two hours and 24 hours (ANCOVA with Tukey contrasts; adj. p< 0.05) (Fig 2A). Hoechst 33342 efflux increased with time at all microfilarial concentrations (lower RFUs) and was marked when 300 and 500 microfilariae were used (Fig 2B). Correlations between the microfilariae concentrations and incubation times were negative due to the decreasing florescence resulting from the efflux of the Hoechst 33342 from within microfilariae. The negative correlations were higher when 300 or 500 microfilariae were used (Fig 2B). Overall differences were not significant between the two strains (ANCOVA with Tukey contrasts; adj. p>0.05). In contrast to resazurin reduction however, differences were significant between microfilariae concentrations for all pairwise comparisons (ANCOVA with Tukey contrasts; adj. p<0.001), except the pairwise comparison of 50 microfilariae and 100 microfilariae (ANCOVA with Tukey contrasts; adj. p>0.05).

### Mode of detection—Resazurin reduction—Fluorescence versus absorbance

Besides fluorescence, resazurin and resorufin can also be colorimetrically detected using absorbance. In this study, absorbance was measured for resazurin (605nm) and resorufin (573nm) at four microfilarial concentrations of JYD-34 and Big Head strain at four incubation times—10 minutes, 1 hour, 2 hours or 24 hours. We hypothesized that absorbance values of resazurin (added dye) would decrease with time, while the absorbance of resorufin (end product) would increase with time. Raw absorbance values of resorufin (S3A and S3B Fig in S1 File) increased with incubation time while absorbance of resazurin (S3C and S3D Fig in S1 File) increased or decreased with variability observed between different microfilariae concentrations.

**Fig 3:**
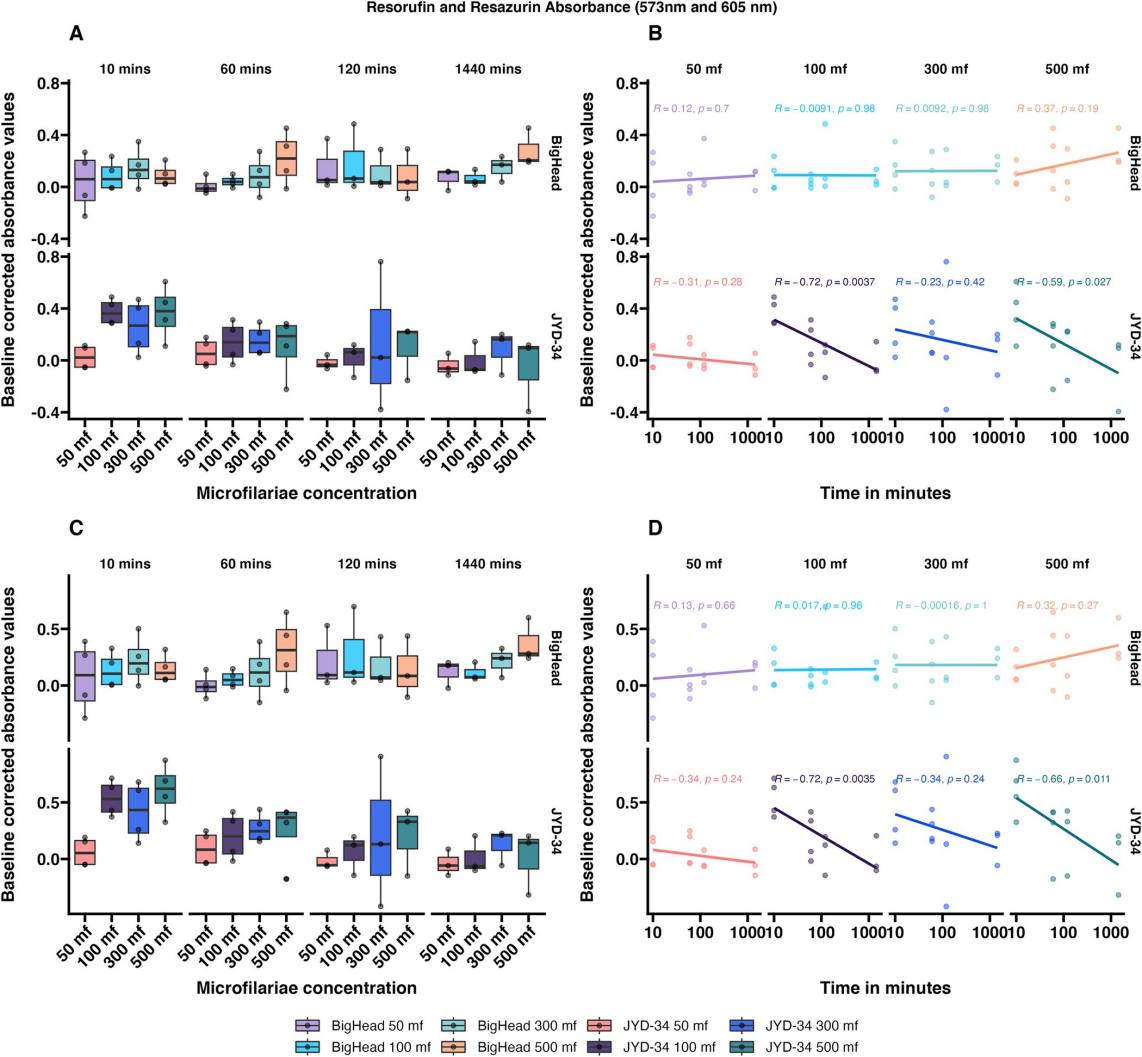
Baseline corrected absorbance values of resorufin (panels A and B) and resazurin (panels C and D) (mean ± SE) obtained by incubating resazurin with *D*. *immitis* microfilariae of two different strains (JYD-34 and Big Head) at four different concentrations (50 to 500 microfilaria per well) for incubation periods ranging from 10 minutes to 24 hours at 37°C. Panel A and C: Values arranged by incubation time. Panel B and D. Values arranged by microfilariae concentration. Correlation values and p-values are shown in panel B.

When controlled for baseline resorufin absorbance (573nm), in overall comparisons, there were no differences observed between strains, microfilarial concentrations or incubation time (ANCOVA with Tukey contrasts; adj. p>0.05) (Fig 3A & 3B). When controlled for resazurin absorbance (605nm), in overall comparisons, there were no differences observed between strains or time (Fig 3C & 3D) (ANCOVA with Tukey contrasts; adj. p>0.05). However, microfilarial concentrations of 50 microfilariae were significantly different from 500 microfilariae (ANCOVA with Tukey contrasts; adj. p<0.01).

### Microfilariae concentration parameter

**Fig 4:**
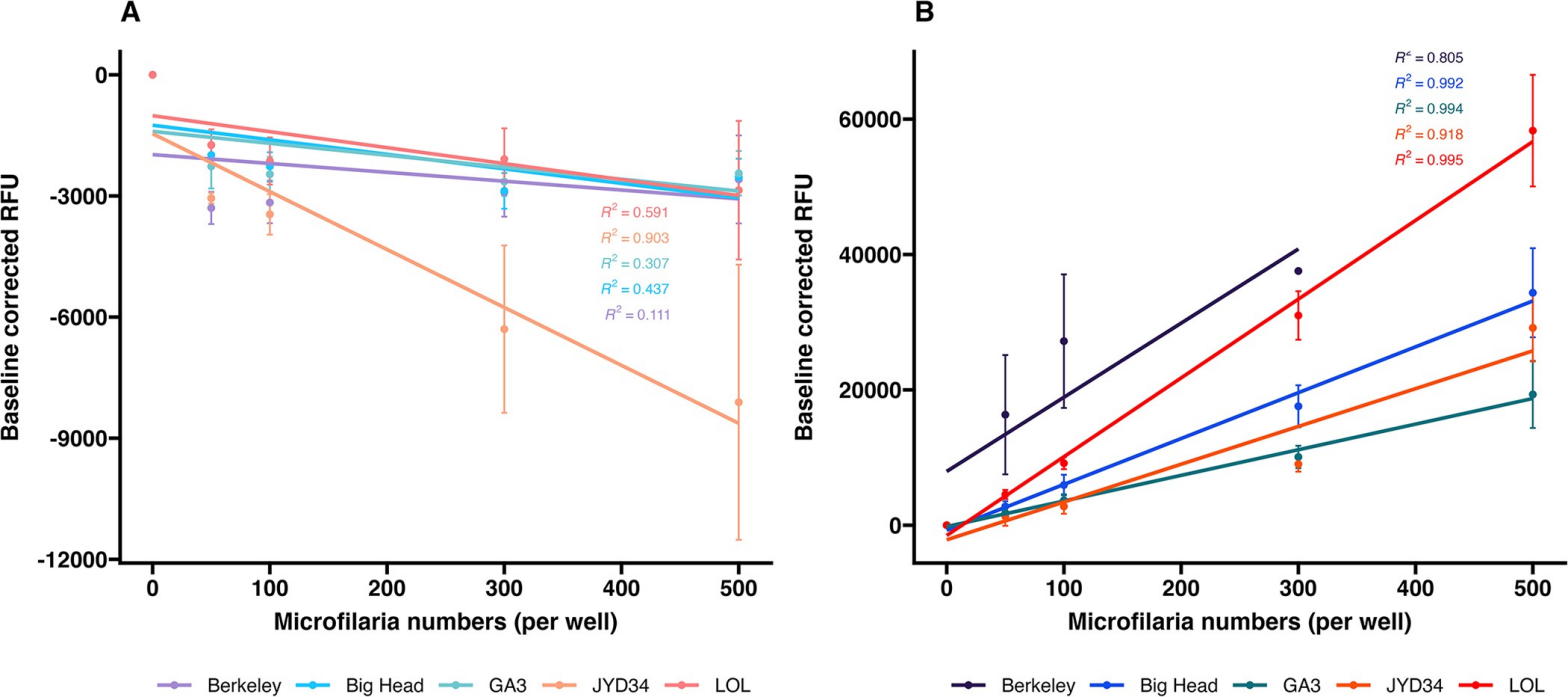
Baseline corrected fluorescence values (mean ± SE) obtained by incubating Resazurin (Panel A) and Hoechst 33342 (Panel B) with *D*. *immitis* microfilariae of five strains (Berkeley, Big Head, GA3, JYD34 and LOL) at four different concentrations (50 to 500 microfilaria per well) for 1 hour at 37°C. Due to low microfilaremia, 500 microfilaria per well could not be tested for Berkley. The coefficient of determination for the linear models (R^2^) is shown.

To test resazurin reduction and Hoechst 33342 efflux, fluorescence was further measured in 3 other *D*. *immitis* strains–Berkeley, Georgia III and LOL, in addition to the two strains used in optimization studies, at 4 microfilarial concentrations (Fig 4).

Resazurin reduction assay: When controlled for baseline resazurin dye fluorescence, in overall comparisons, there were significant differences in resazurin reduction between JYD-34 and Big Head (ANCOVA with Tukey contrasts; adj. p<0.001), JYD-34 and Georgia III (ANCOVA with Tukey contrasts; adj. p<0.01) and JYD-34 and LOL (ANCOVA with Tukey contrasts; adj. p<0.001) (Fig 4A). No significant differences were found between microfilarial concentrations (ANCOVA with Tukey contrasts; adj. p>0.05).

Hoechst 33342 efflux assay: When controlled for baseline dye fluorescence, in overall comparisons, there were significant differences in Hoechst 33342 efflux between (i) Berkeley and three other strains–Big Head, Georgia III and JYD-34 (ANCOVA with Tukey contrasts; adj. p<0.001) and (ii) between LOL and three other strains—Big Head (p<0.01), Georgia III (p<0.001) and JYD-34 (p<0.001) (Fig 4B). There were also significant differences at all pairwise microfilarial concentrations (ANCOVA with Tukey contrasts; adj. p<0.001), except the pairwise comparison of 50 microfilariae and 100 microfilariae (ANCOVA with Tukey contrasts; adj. p>0.05). Due to low microfilaremia, 500 microfilaria per well could not be tested for Berkley.

### Effect of ivermectin and doxycycline

**Fig 5:**
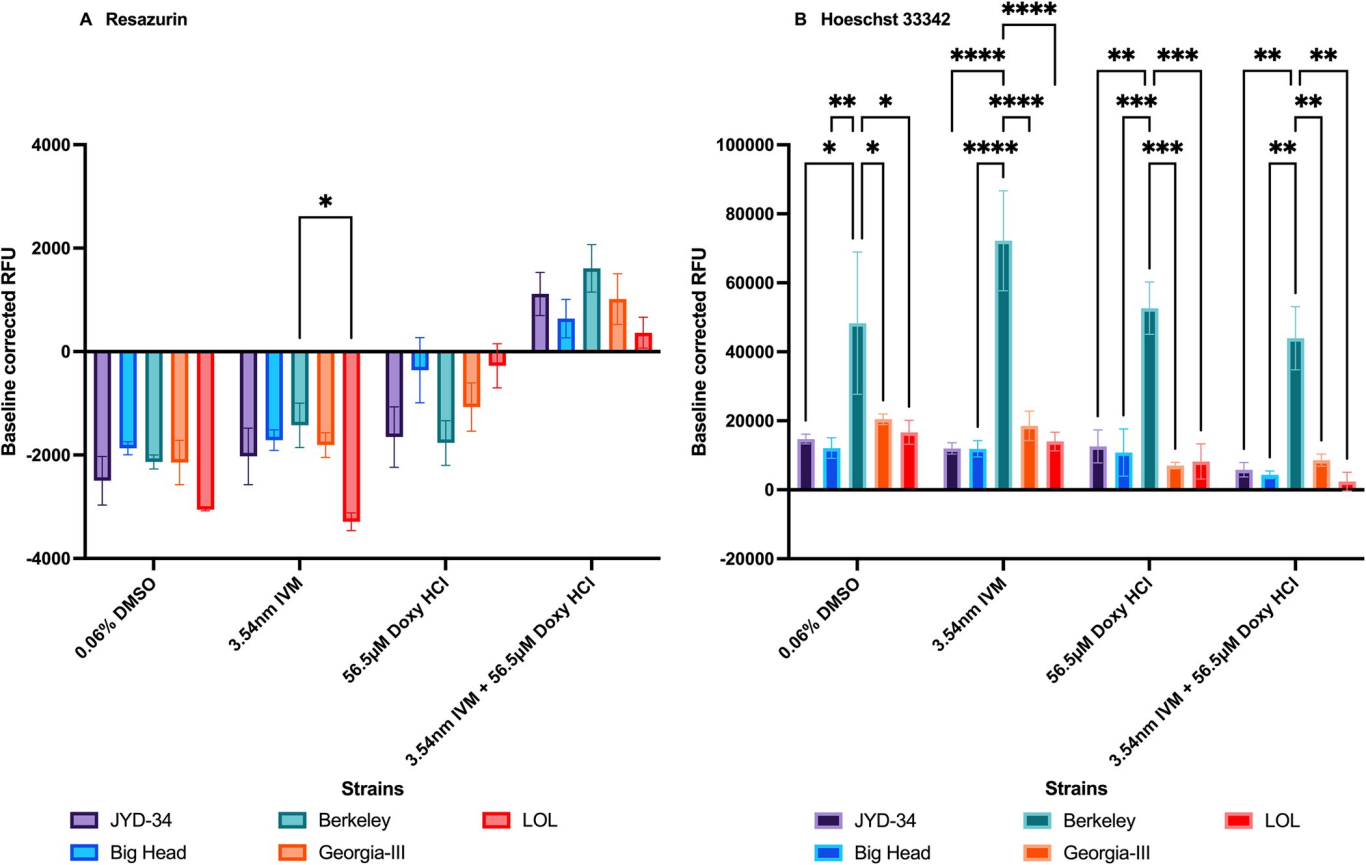
Baseline corrected fluorescence values (mean ± SE) obtained by incubating drugs with 300 *D*. *immitis* microfilariae of 5 strains for 1 hour, followed by incubation with resazurin (Panel A) or Hoechst 33342 (Panel B) for 1 hour. Two-way ANOVA with Tukey’s multiple comparison was performed. Only comparisons between strains for each treatment are shown.

To determine the effects of ivermectin and doxycycline on the assays, *D*. *immitis* microfilariae were incubated with ivermectin alone, doxycycline alone or a combination of ivermectin and doxycycline and fluorescence was recorded. Microfilariae of each strain exposed to 0.06% DMSO was used as control. In the resazurin reduction assay, after baseline correction, the effect of doxycycline and ivermectin in combination caused significant reduction of resazurin to resorufin in most strains compared to the two drugs alone (Two way ANOVA; p<0.05) (S5 Fig in S1 File). Between strain differences were seen between Berkeley and LOL (Two-way ANOVA; p<0.05) (Fig 5).

In the Hoechst 33342 efflux assay, after baseline correction, significant levels of between strain differences were observed in each treatment (Two-way ANOVA; p<0.05) (Fig 5). There were also significant differences in the Berkeley microfilariae exposed to ivermectin alone and Berkeley microfilariae exposed to doxycycline alone (Two-way ANOVA; p<0.05) (S5 Fig in S1 File).

### Relative expression of candidate genes that govern the assays

**Fig 6:**
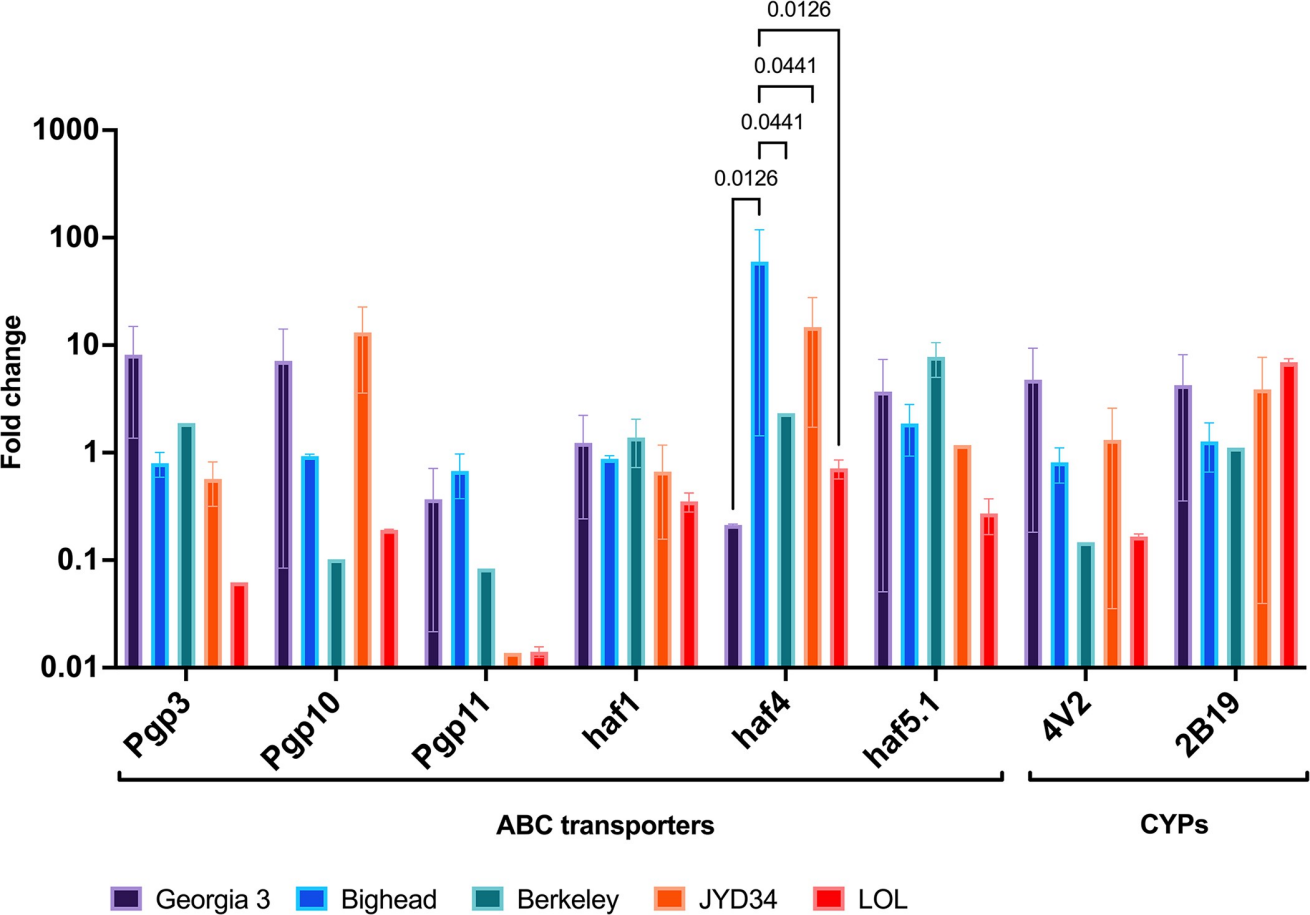
Relative expression of P-glycoprotein and cytochrome P450s measured using qPCR. Two-way ANOVA with false discovery rate correction was performed. q-values are reported.

Constitutive expression levels of ABC transporters—*Pgp* and *haf* transporters and cytochrome P450 genes were determined using qPCR in *D*. *immitis* microfilariae (Fig 6). Expression of three Pgp genes was detected in in all 5 strains, but expression did not differ significantly between the strains tested (Two-way ANOVA with FDR correction; q-value>0.05). Expression of three other ABC transporters *Dim-haf-1*, *Dim-haf-4* and *Dim-haf-5*.*1* were also detected in all strains tested. Expression of *Dim-haf-4* was significantly higher in the Big Head strain compared to all the other strains tested (Two-way ANOVA with FDR correction; q-value<0.05). Expression of two cytochrome P450 genes were also found in all strains tested with no differences in expression (Two-way ANOVA with FDR correction; q-value>0.05).

## Discussion

With the emergence of macrocyclic lactone resistance in *Dirofilaria immitis* within the U.S., there has been a need for diagnostic tests to distinguish susceptible and resistant isolates. The development of such a robust, inexpensive, quick test requires significant research with a wide range of known validated isolates. However, constitutive variations within and between groups of *D*. *immitis* isolates that have been designated “resistant” or “susceptible” have not been fully characterized, although genetic variation is beginning to be studied [13]. Since *D*. *immitis* is a complex eukaryotic organism, populations of worms should be expected to have inherent constitutive variation. Such constitutive variation is manifested at the strain-level in prokaryotes [31] and is of importance in host-pathogen/cross-species interactions, as demonstrated between *C*. *elegans* strains and its gut microbiome [32]. In this study we show that microfilariae of *D*. *immitis* shows between -strain variations in two biochemical assays. This knowledge is important for the development of *in vitro* assays to differentiate susceptible and resistant strains in the future.

Metabolism within organisms is mediated by several pathways involving multiple genes and proteins. Biochemically, redox reactions are one type of metabolic reactions that occur during homeostasis. Redox reactions can be measured using assays such as resazurin reduction. In a previous study we had shown that resazurin reduction varied in different *D*. *immitis* microfilarial isolates, and was likely associated with differences in basal metabolic levels [18]. However, only one concentration and time-point of resazurin reduction by *D*. *immitis* microfilariae had been tested in four strains (JYD-27, Yazoo, Metairie, Missouri). In this study, we determined the effects of several parameters such as incubation time, microfilarial density in assay plate wells and the mode of detection on the tests. We further expanded testing to five other strains. We observed that resazurin reduction to resorufin measured as the fluorescence of resorufin did not significantly change over time or with microfilarial concentrations (Fig 1). Absorbance values of resazurin and resorufin recorded using a colorimetric spectrophotometer failed to show any differences between strains, incubation times or microfilarial densities (Fig 3). However, when an optimized concentration of 300 microfilariae/well was tested with a 1-hour incubation period using a fluorescent spectrophotometer, we observed strain-level variations of assay readouts in the absence of drugs (Fig 4). Interestingly, there were significant differences in resazurin reduction between JYD-34 (a resistant isolate), two susceptible strains (Georgia III, Big Head) and one unknown strain (LOL) but not with the Berkeley (susceptible) strain. This is likely due to differences in metabolism between naturally occurring isolates of the *D*. *immitis*; however, further studies on microfilarial metabolism are necessary to determine if metabolism affects ML-associated phenotype.

ATP binding cassette (ABC) transporters have been studied in the context of *D*. *immitis* resistance [10,25,33–35]. One of the two SNPs that show the highest association with clinical resistance [12] is located in a P-glycoprotein (P-gp; ABCB1) gene. Additionally, these transporters have been implicated in anthelmintic resistance in other nematodes [36–38]. Thus, P-glycoproteins and ABC transporters of parasitic nematodes are candidates of interest in macrocyclic lactone resistance. Dye efflux mediated through the ABCB1 transporters can be measured using the Hoechst 33342 assay [18]. The fluorescent dye Hoechst 33342 is a P-gp substrate, fluoresces only when associated with biological membranes and does not fluoresce in aqueous solutions. This property makes the dye convenient for fluorescence-based assays. The disposition and interaction between Hoechst 33342 and nematode P-glycoprotein has been demonstrated in heterologous expression systems [39] and in whole worm larval stages [40]. We had also previously shown that basal levels of P-gp efflux activity varied between *D*. *immitis* strains [18]. We demonstrated that Hoechst 33342 fluorescence within microfilariae shows a negative linear relationship with incubation time (Fig 2), due to Pgp-mediated removal of the dye from the microfilariae. When a single incubation time-point was used, Hoechst 33342 retention increased with increasing numbers of microfilariae (Fig 4). Overall, two susceptible strains—Berkeley and LOL retained the highest amount of dye and thus had the lowest amount of P-gp mediated efflux (Fig 4). Thus, naturally occurring isolates likely have variable P-gp activity.

We tested the effect of the drugs ivermectin, doxycycline or a combination of the two on assay read-outs in the two tests. These drugs were chosen to mimic their effect in *D*. *immitis* treatment regimens as recommended by the American Heartworm Society. The concentrations and times tested in this study reflect pharmacokinetic Cmax in plasma in agreement with previous studies [25]. Strain level variation was minimal in the resazurin study (Fig 5A). Resazurin reduction was significantly higher in the presence of doxycycline and ivermectin than ivermectin or doxycycline alone (S5 Fig in S1 File). Synergistic effects of the combination on microfilarial metabolism may be the cause of this observation since the drugs did not significantly alter metabolism individually. In the Hoechst 33342 study, between strain variation was observed across treatment groups; however treatments had low effects on competitive efflux (Fig 5B). The contribution of *Wolbachia* within microfilariae to the outcomes of the two assays is currently unknown. Much is also unknown about the effects of drugs on microfilarial metabolism and ABC transporter mediated efflux. Since resistance is associated with ABC transporters in *D*. *immitis* [10,12,41], the effect of the treatment on these transporters and other pathways must be studied further to leverage them for microfilarial control.

Since the assays tested in this study were based on metabolism and efflux, the transcription of candidate genes associated with these functions were studied (Fig 6). The ABC transporter family is greatly expanded in number in a parasitic nematodes [42] compared to mammals. In *D*. *immitis*, three P-glycoprotein genes (ABCB1) and three *haf* transporters (ABCB and ABCC) have been described [34]. This number is lower that the repertoire of P-gps in filarial nematode– 8 in *Brugia malayi* [43], and P-gp gene repertoires in other Clade III nematodes– 10 in *Parascaris univalens* [44] and 10 in *Toxocara canis* [40]. The detection of only a few transporters is likely to be artefactual due to the incomplete and fragmented nature of all publicly available *D*. *immitis* genomes, which make gene annotation and genome mining difficult. Thus, the need for chromosome-level genome and transcriptome assemblies of multiple strains of *D*. *immitis* is acute. Differences in constitutive expression of ABC genes have been studied in *D*. *immitis* adults [25]. We demonstrated that individual ABC genes have strain-level variable expression in *D*. *immitis* microfilariae, although these transcriptional differences were not statistically significant. The contributions of *Dim-Pgp-11* to macrocyclic lactone efflux [41] has been studied but the magnitude of the effect of other Pgp genes are not fully understood. Our understanding of the effects of the whole repertoire of P-gps and other ABC transporters on ML resistance in *D*. *immitis* is incomplete and needs further study. Only three P-gps and three *haf* transporters have been described in *D*. *immitis* [34]. Since other nematodes in clade III have larger ABCB repertoires (16 in *Brugia malayi* [43], 10 in *Parascaris univalens* [44] and 13 in *Toxocara canis* [40]), it is expected that other transporters in *D*. *immitis* will be identified when the genome quality and annotation improves.

Cytochrome P450 oxidoreductases (CYPs) play a role in redox reactions and drugs detoxification in parasites and may serve as potential drug targets [45]. CYPs have not been well-studied in clade III nematodes. We used orthologous data from *Haemonchus contortus* [46] to identify CYPs in the *D*. *immitis* genome. We also showed that strain-level differences in CYP expression exists despite not being statistically significant (Fig 6). The effect of breed level differences in CYPs are known to affect drug metabolism in dogs [47]. The effect of strain-level differences in *D*. *immitis* on drug metabolism must be studied in the future.

Some variables that were not assessed in this study were (i) the effects of chilling of the blood at 4°C for transport and storage, and (ii) the effects of different microfilarial isolation methods on the outcomes of the assays. While cryopreserved *D*. *immitis* microfilariae can be reactivated to complete the lifecycle [48], the effect on blood storage at 4°C is not fully understood. It is unknown if different strains of *D*. *immitis* have differences in cold tolerance and how metabolism is affected by storage temperatures. Isolation methods such as saponin and sodium citrate-based methods were shown to have an effect on motility assays [15]. These variables should be assessed in future studies.

The development of the perfect assay to differentiate or predict drug resistance and susceptibility has been thwarted by strain-level differences, which may cause *in vitro* assay outputs to differ from clinical phenotypes observed using MFST. These strain-level differences may be due to the genetic composition, diversity and epigenetic variation, which have not been studied yet. The exact genetic relationships between microfilariae, L3s and the parental population of *D*. *immitis* from which they originate and the impact that such genetic diversity has on the resistant phenotype of the worm is not completely understood. The differential effect of drugs on L3s and microfilaria in vitro, without the influence of the host immune system is not clearly understood. In a genotyping study of *D*. *immitis* microfilariae, different passages of laboratory strains originating from the same source of infection passed in different dogs generated microfilariae that were genetically diverse [13]. The diverse infrapopulation found was explained by the polyandrous nature of parental worms in infections with high worm burdens. Interestingly, it was found that microfilaricidal treatments did not alter the genetic composition of *D*. *immitis* microfilarial infrapopulations [13]. However, in the related helminth *Wuchereria bancrofti*, drug treatments were theorized to increase infrapopulation genetic heterogeneity [49]. Thus, the exact reason for strain-level variations is not completely understood or characterized yet.

## Conclusions

In conclusion, data from this study suggests that strain-level variations in *D*. *immitis* may be significant and these variations are detectable in biochemical assays of metabolism and ABC transporter mediated efflux. Strain-level variations in these assays may reflect the inherent complex nature of genomic, transcriptomic and proteomic variations within the worms despite clinical phenotype classifications of these strains into broad “ML-susceptible” and “ML-resistant” categories. Further studies into strain-level variation are needed to develop an integrated, systems-level understanding of *D*. *immitis* and resistance to ML drugs.

## Supporting information

S1 File Contains supporting figures. (DOCX)

## Abbreviations

L3: Third larval stage
iL3: Infective third larval stage
qPCR: Qualitative Polymerase Chain Reaction
ML: Macrocyclic lactone
ABC: transporter: ATP binding cassette transporter
P-gp: Permeability glycoprotein
CYP: Cytochrome P450
