# Supplementary material for: Strain-level variations of Dirofilaria immitis microfilariae in two biochemical assays
Source: PLoS One. 2024 Jul 17;19(7):e0307261. doi: 10.1371/journal.pone.0307261 (PMC11253964; doi:10.1371/journal.pone.0307261)
Supplement: S1 File — (DOCX) [file pone.0307261.s001.docx]

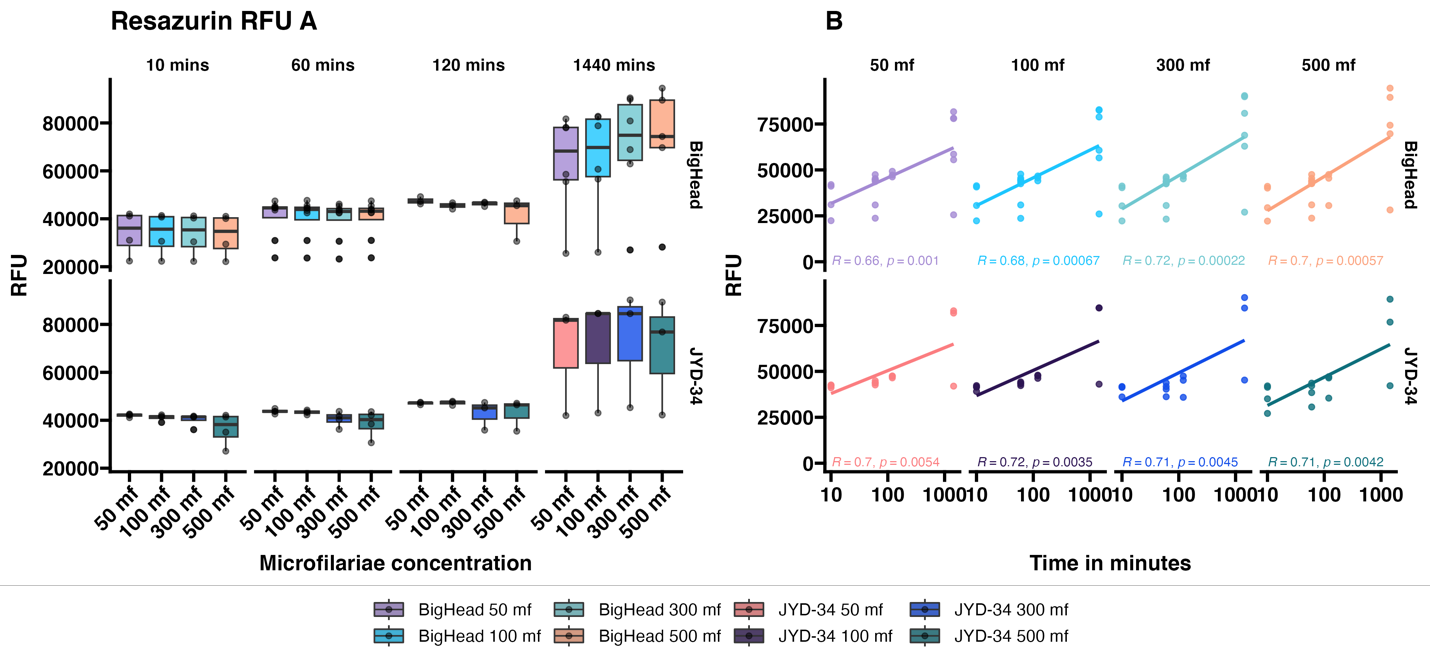


**Fig. S1.** Raw relative fluorescence units (RFU) obtained by incubating resazurin with *Dirofilaria immitis* microfilariae of two different strains (JYD-34 and BigHead) at four different concentrations (50 to 500 microfilaria per well) for different incubation periods (10 minutes to 24 hours). Panel A: Values arranged by incubation time. Panel B. Values arranged by microfilariae concentration.


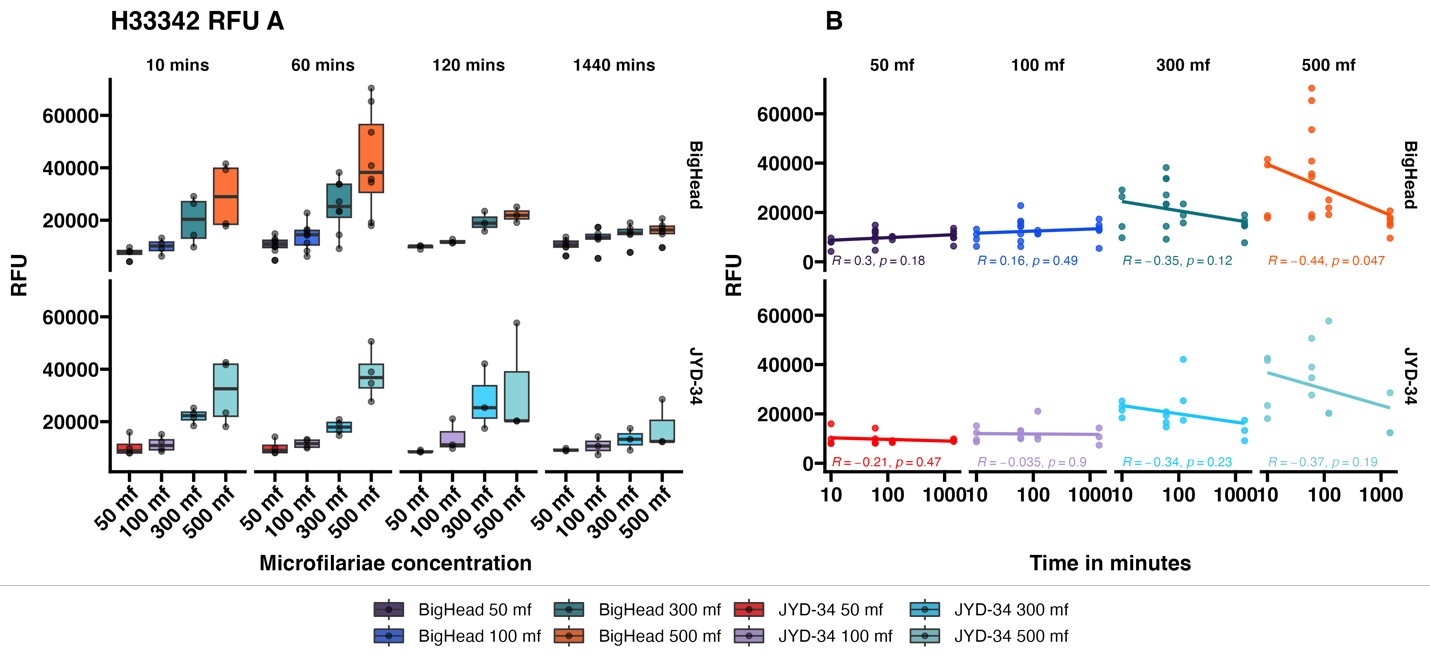


**Fig. S2.** Raw relative fluorescence units (RFU) obtained by incubating Hoechst 33342 with *Dirofilaria immitis* microfilariae of two different strains (JYD-34 and BigHead) at four different concentrations (50 to 500 microfilaria per well) for different incubation periods (10 minutes to 24 hours). Panel A: Values arranged by incubation time. Panel B. Values arranged by microfilariae concentration.


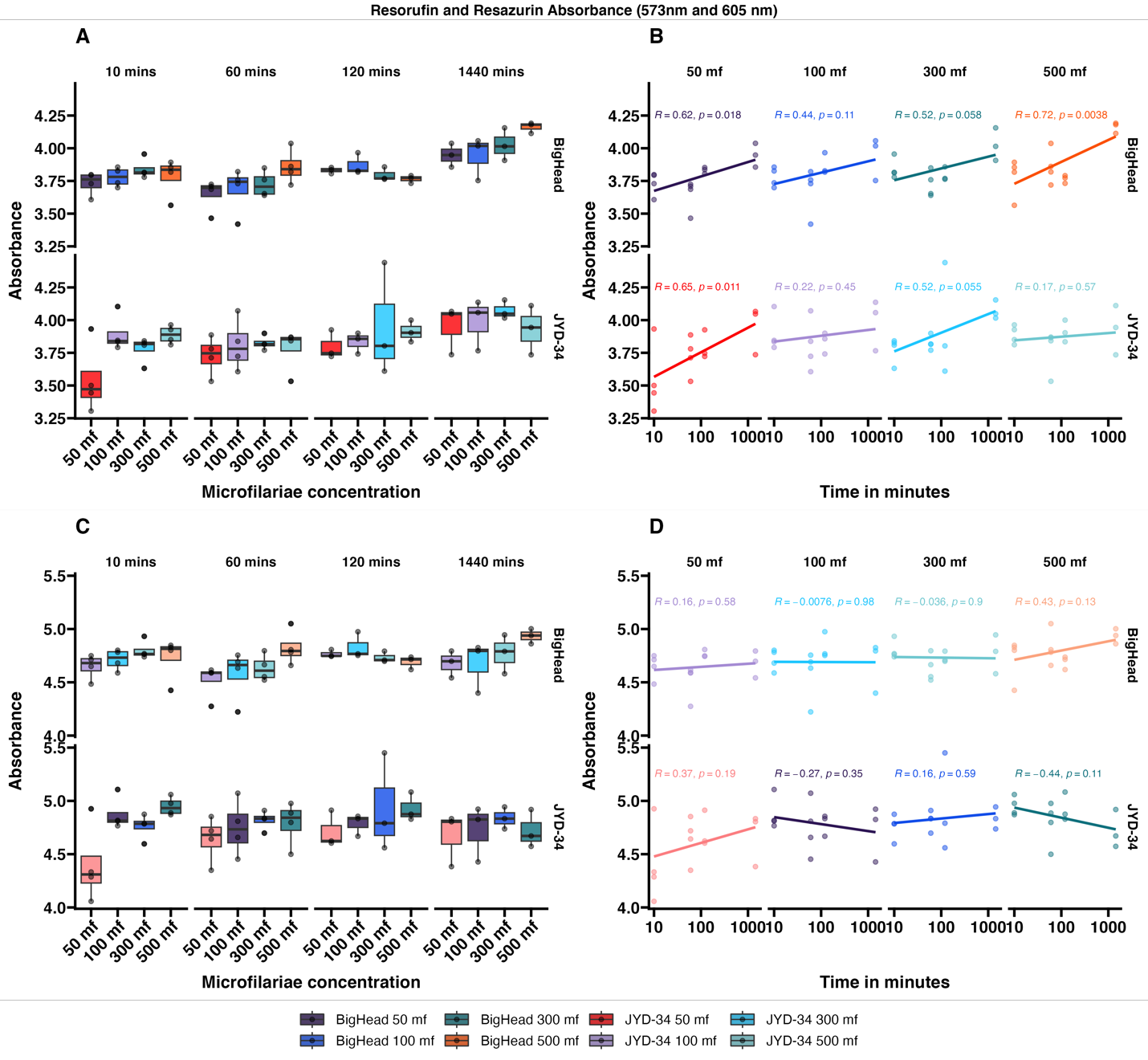


**Fig. S3.** Raw absorbance values of resorufin (panels A and B) and resazurin (panels C and D) obtained by incubating resazurin with *Dirofilaria immitis* microfilariae of two different strains (JYD-34 and BigHead) at four different concentrations (50 to 500 microfilaria per well) for incubation periods ranging from 10 minutes to 24 hours at 37$^{\circ}$C. Panel A and C: Values arranged by incubation time. Panel B and D. Values arranged by microfilariae concentration.


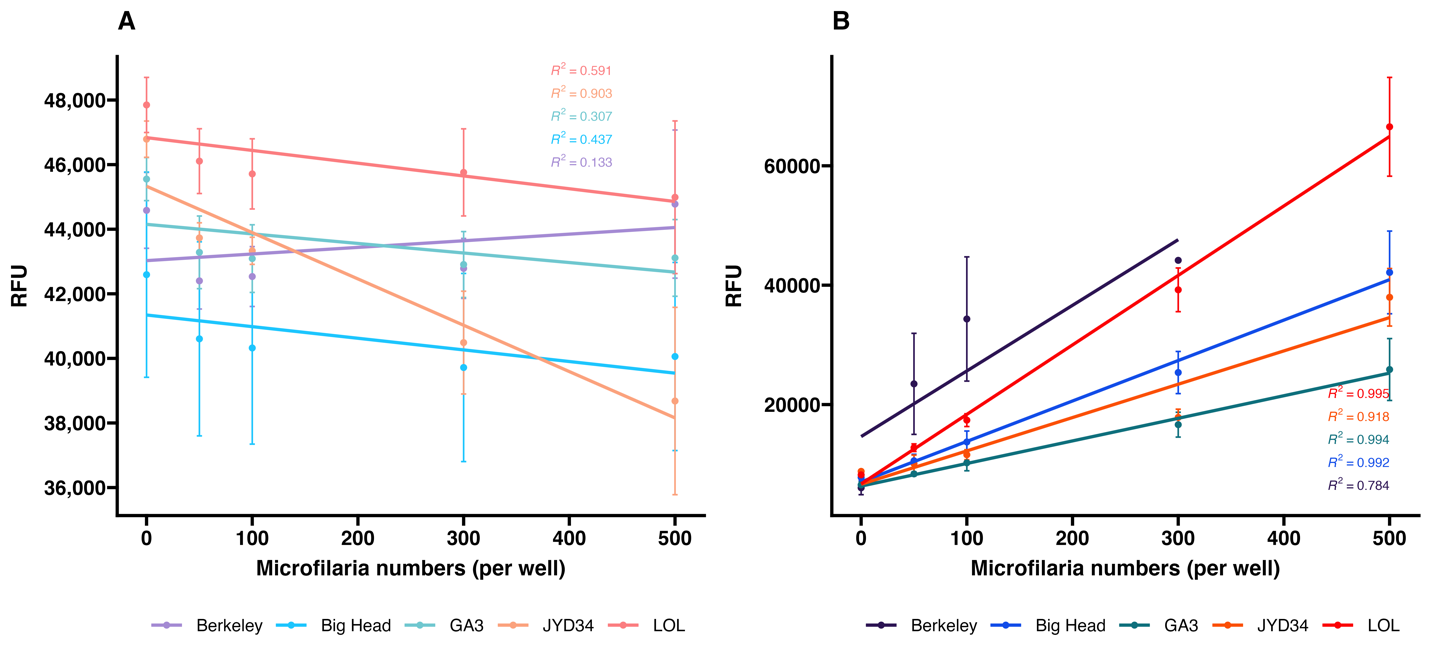


**Fig. S4.** Raw relative fluorescence units (RFU) obtained by incubating Resazurin (Panel A) and Hoechst 33342 (Panel B) with *Dirofilaria immitis* microfilariae of five strains (Berkeley, Big Head, GA3, JYD34 and LOL) at four different concentrations (50 to 500 microfilaria per well) for 1 hour at 37$^{\circ}$C.

**Fig. S5.** Baseline corrected fluorescence values (mean ± SE) obtained by incubating drugs with 300 *D. immitis* microfilariae of 5 strains for 1 hour, followed by incubation with resazurin. Two-way ANOVA with Tukey’s multiple comparison was performed. Pairwise comparisons with p <0.001 are shown.

**Fig. S6.** Baseline corrected fluorescence values (mean ± SE) obtained by incubating drugs with 300 *D. immitis* microfilariae of 5 strains for 1 hour, followed by incubation with Hoechst 33342. Two-way ANOVA with Tukey’s multiple comparison was performed. Pairwise comparisons with p <0.001 are shown.
